# Subchronic Toxicity of Copper Oxide Nanoparticles and Its Attenuation with the Help of a Combination of Bioprotectors

**DOI:** 10.3390/ijms150712379

**Published:** 2014-07-14

**Authors:** Larisa I. Privalova, Boris A. Katsnelson, Nadezhda V. Loginova, Vladimir B. Gurvich, Vladimir Y. Shur, Irene E. Valamina, Oleg H. Makeyev, Marina P. Sutunkova, Ilzira A. Minigalieva, Ekaterina P. Kireyeva, Vadim O. Rusakov, Anastasia E. Tyurnina, Roman V. Kozin, Ekaterina Y. Meshtcheryakova, Artem V. Korotkov, Eugene A. Shuman, Anastasia E. Zvereva, Svetlana V. Kostykova

**Affiliations:** 1 The Medical Research Center for Prophylaxis and Health Protection in Industrial Workers, 30 Popov Str., Ekaterinburg 630014, Russia; E-Mails: privalovali@yahoo.com (L.I.P.); tushkanN@yandex.ru (N.V.L.); Gurvich@ymrc.ru (V.B.G.); marinasutunkova@yandex.ru (M.P.S.); minigalieva@yandex.ru (I.A.M.); katerinakir@yandex.ru (E.P.K.); honored_sci@yahoo.com (V.O.R.); 2 The Institute of Natural Sciences, the Ural Federal University, Ekaterinburg 630000, Russia; E-Mails: vladimir.shur@usu.ru (V.Y.S.); anastasiya.tyurnina@labfer.usu.ru (A.E.T.); roman.kozin@labfer.usu.ru (R.V.K.); 3 Central Research Laboratory, the Ural State Medical University, 17 Klyuchevskaya Str., Ekaterinburg 630109, Russia; E-Mails: ivalamina@mail.ru (I.E.V.); ommt305@mail.ru (O.H.M.); katusha-ugma@rambler.ru (E.Y.M.); akorotkov64@mail.ru (A.V.K.); eshuman@gmail.com (E.A.S.); dor_3110@mail.ru (A.E.Z.); svkostyukova@mail.ru (S.V.K.)

**Keywords:** copper oxide nanoparticles, subchronic toxicity, genotoxicity, bioprotectors

## Abstract

In the copper metallurgy workplace air is polluted with condensation aerosols, which a significant fraction of is presented by copper oxide particles <100 nm. In the scientific literature, there is a lack of their *in vivo* toxicity characterization and virtually no attempts of enhancing organism’s resistance to their impact. A stable suspension of copper oxide particles with mean (±SD) diameter 20 ± 10 nm was prepared by laser ablation of pure copper in water. It was being injected intraperitoneally to rats at a dose of 10 mg/kg (0.5 mg per mL of deionized water) three times a week up to 19 injections. In parallel, another group of rats was so injected with the same suspension against the background of oral administration of a “bio-protective complex” (BPC) comprising pectin, a multivitamin-multimineral preparation, some amino acids and fish oil rich in ω-3 PUFA. After the termination of injections, many functional and biochemical indices for the organism’s status, as well as pathological changes of liver, spleen, kidneys, and brain microscopic structure were evaluated for signs of toxicity. In the same organs we have measured accumulation of copper while their cells were used for performing the Random Amplification of Polymorphic DNA (RAPD) test for DNA fragmentation. The same features were assessed in control rats infected intraperitoneally with water with or without administration of the BPC. The copper oxide nanoparticles proved adversely bio-active in all respects considered in this study, their active *in vivo* solubilization in biological fluids playing presumably an important role in both toxicokinetics and toxicodynamics. The BPC proposed and tested by us attenuated systemic and target organs toxicity, as well as genotoxicity of this substance. Judging by experimental data obtained in this investigation, occupational exposures to nano-scale copper oxide particles can present a significant health risk while the further search for its management with the help of innocuous bioprotectors seems to be justified.

## 1. Introduction

The actively developing branch of particle toxicology known as “nanotoxicology” attaches a special importance to assessing the effects of metal and metal-oxide nanoparticles. It is known that the workroom and ambient air in metallurgical, welding, and some chemical technologies may contain condensation aerosols with particles of sizes falling within the submicron (including, nanometer) range, while the chemical composition, being presumably metal oxides, cannot be always accurately determined (this applies particularly to mixed-valence transition metals). Thus, engineered nanoparticles (NPs) of this kind are of interest for industrial toxicology not only as such, but also as a model object for characterizing the harmful action of the nano-fractions of corresponding industrial aerosols on the organism. Based on our studies of supermagnetic iron oxide (II, III), silver, and gold nanoparticles [1,2,3,4,5,6,7,8] which are actually encountered only in the manufacture of corresponding nanomaterials and in their applications in science, technology, or medicine, we proceeded to experimental research into the here stated problem, starting with copper oxide nanoparticles.

**Figure 1:**
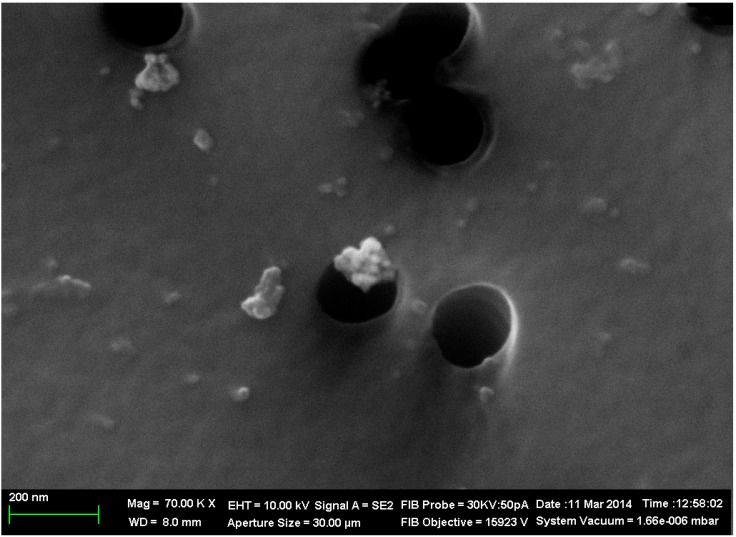
Sinle and aggregated particles sampled on a polycarbonate filter from the ambient air of a cathode copper smelting and casting facility. Scanning electron microscopy, magnification ×70,000 (black spots are filter’s pores).

**Figure 2:**
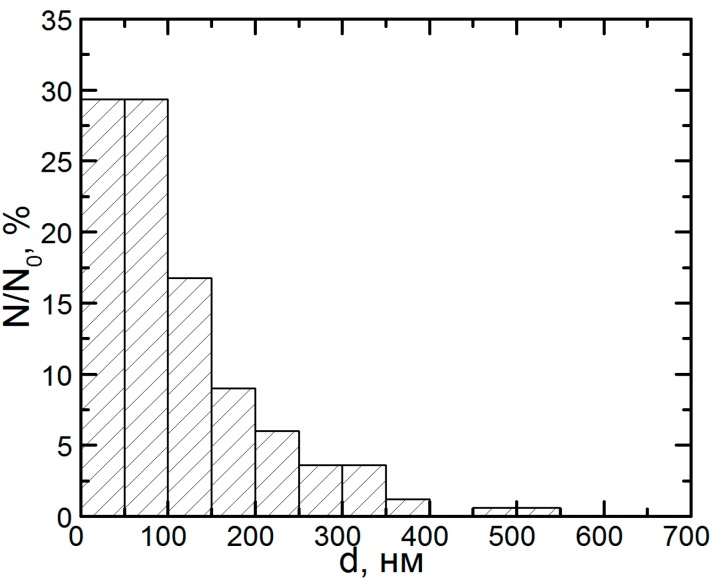
Percentage distribution of particles by size in the submicron range on filters through which the ambient air of a cathode copper smelting and casting facility had been drawn (obtained by a program measuring and counting 500 spherical particles). N—Number of particles of a given diameter; N_0_—Total number of particles.

Figure 1 and Figure 2 show the substantial contribution of spherical particles with diameter <100 nm or their aggregates in samples gathered with the help of polycarbonate micropore filters from the ambient air in workshops where the smelting and casting of refined (cathode) copper take place [9]. The X-ray microanalysis has made it possible to identify the chemical composition of large (300–500 nm) nanoparticle aggregates, which were found to contain Cu and O in an atomic ratio of approximately 1:1. This, however, does not mean that they consist of copper oxide СuO since particles, as well as condensed phases in general, that form in the course of vapor condensation, typically display considerable deviations from stoichiometry. Quite probably, these nanoparticles consist of a mixture of various copper oxides, which cannot be identified as a certain chemical compound.

For their toxicological characterization, in our experiments we used nanoparticles of the same atomic composition specially obtained by the method of laser ablation of pure copper as described in Section Methods.

Thus, toxicological characterization of copper oxide nanoparticles is directly relevant to research into occupational health risks at copper smelters and copper refineries. Although such characterization had to rely, first of all, on the considerable number of published studies, their review showed that some essentially important questions of copper nanotoxicology cannot be considered as resolved.

It is indicative that the very first of such articles [10] had the title starting with the words “Copper oxide nanoparticles are highly toxic”. These authors carried out experiments on a stable human lung epithelial cell line (А549) and compared the effects of CuO, TiO_2_, ZnO, CuZnFe_2_O_4_, Fe_3_O_4_, and Fe_2_O_3_ nanoparticles with those of carbon nanotubes on cell viability (by trypan blue exclusion), production of reactive oxygen species and DNA damage and found CuO nanoparticles to be most potent in all three indices. The authors maintained that it could not be explained by the release of Cu-ions in the cell medium. On the contrary, Bondarenko *et al.* [11] compared the action of CuO nano- and micro-particles on Coli bacteria with their solubility and with the effect of CuSO_4_ and came to the conclusion that it is the dissolution of CuO particles that is the key factor triggering the impacts on the above indices. Pang *et al.* [12] did not find any quantitative differences in the effects of CuCl_2_, CuO nano- (6 and 100 nm) and micro-particles (<5 µm) on deposit-feeding snails (*Potamopygus antipodarum*), for a fixed concentration of copper, which provides indirect evidence in favor of the key role of Cu ion.

Limbach *et al.* [13], who unequivocally entitled their article as “Nanoparticle cytotoxicity depends on intracellular solubility, *etc.*”, compared the loss of viability by established cell lines (human HeLa and Chinese hamster oocytes) under exposure to elemental copper nanoparticles coated with carbon and thus rendered almost insoluble, or to CuO nanoparticles easily dissolving in model buffers, and found the latter to be much more cytotoxic at equal dosage. Ionic copper had virtually the same effect as CuO nanoparticles.

Cronholm *et al.* [14], who also used established human lung cell lines (А549 and BEAS-2B), showed that, unlike copper ions, nanoparticles are captured by cells, but they attributed their findings of cytotoxic effect and DNA damage to the release of Cu ions following intracellular dissolution of nanoparticles. The authors did not find nanosilver to be cytotoxic and genotoxic, explaining this by low release of Ag-ions within short time periods. It should be noted, however, that the cytotoxicity and genotoxicity of nanosilver has been demonstrated in many experimental models referred to in our paper [7], presenting our own estimates of these effects *in vivo* as well, which are positive beyond any doubt.

Сuillet *et al.* [15] used hepatocyte cell lines HepG2 in their experiments to compare the action of subtoxic doses of СuO nanoparticles and CuCl_2_ in equivalent doses of copper on the expression of some genes encoding proteins participating in the intracellular homeostasis of copper and zinc, and on the direct indices of that homeostasis. According to these authors, their findings provide evidence that СuO nanoparticles act as the Trojan horse: They enter hepatic cells (most probably by endocytosis), bypassing the cellular defense mechanisms against excess Cu, but their subsequent endosomal dissolution leads to an increase in the Cu concentration inside the hepatocyte.

Based on a review of extensive literature, Fröhlich [16] believes that, commonly for many metals and their oxides, the release of free ions is a second mechanism of nanoparticle cytotoxicity (the first one being the generation of reactive oxygen species as a result of various chemical reactions); however, the author does not mention nano-Cu oxide in this context.

It should be emphasized that, to the best of our knowledge, there are no publications that would estimate not only the toxicodynamic but also the toxicokinetic role of CuO nanoparticle solubility in a whole body experiment on laboratory mammals subjected to chronic or, at least, subchronic exposure. Meanwhile, not only a priori considerations but also comparison to our data on the toxicokinetics of other metal nanoparticles, the solubility of which was different due to differences in size for the same chemical composition as in the case of magnetite [2,3], or due to the chemical nature of the metal as in the case of comparison of equidimensional silver and gold nanoparticles [7], suggested that in a long-term exposure it is this property of nanometals that controls the degree of their retention in internal organs and, thus, their target organ toxicity.

Copper nanoparticles were shown to be more aggressive than copper microparticles in relation to acute lethal toxicity for mice [17], kidney, liver, and spleen being the primary target organs, and to subacute nephrotoxic effect for rats [18].

From the above, it can be seen that DNA damage due to copper oxide nanoparticles was observed already in early research into this area. Moreover, Pan *et al.* [19] found in an Ames test that CuO nanoparticles are mutagenic, a feature that Al_2_O_3_, Co_3_O_4_, TiO_2_, and ZnO nanoparticles do not display in similar conditions. Song *et al.* [20] found in a short-term test on mice, that the number of micronuclei in circulating blood reticulocytes increased in response to i.p. injection of CuO, Fe_3_O_4_, Fe_2_O_3_, Fe_2_O_3_, TiO_2_, and Ag nanoparticles; however, only nano-CuO gave a dose-dependent increase in the levels of 8-hydroxy-2'-deoxyguanosine in liver DNA. Gomes *et al.* [21] found DNA damage to be induced in hemolymph cells of the mussel Mytilus galloprovincialis in response to CuO and Ag nanoparticles, ionic forms of these metals being even more genotoxic (suggesting different mechanisms of action, according to the authors). Alarifi *et al.* [22] showed that CuO nanoparticles produce a cytotoxic and genotoxic action on human skin keratocytes *in vitro*, possibly mediated by oxidative stress. Akhtar *et al.* [23], who used the human lung epithelial cell line А549, also showed that 24 nm CuO particles are cytotoxic and genotoxic in a dose-dependent manner as assessed by the comet assay and micronucleus test, both being closely correlated with the generation of reactive oxygen species.

At the same time, the authors of a literature review on the genotoxicity and carcinogenicity of cobalt-, nickel-, and copper-based nanoparticles published in 2012 [24] emphasized that for these, and for nano-copper in particular, there were very few studies *in vivo* available.

Finally, we have found virtually no information concerning any attempts to enhance the resistance of the whole organism to toxic and genotoxic impacts of copper or copper oxide nanoparticles. As well as in relation to many other metal particles, some authors associate the toxic effects of copper nanoparticles with oxidative stress induction (in addition to the above-mentioned references, see also [25]); however, we are aware of only one study carried out on isolated hepatocytes to test the protective effect of an anti-oxidant agent, specifically vitamin E, and not in response to nanoparticles but to CuCl_2_ solution at that [26].

The concept of the “biological prophylaxis”, its theoretical premises and general principles, as well as numerous examples of their realization have been published by us repeatedly, including, in review articles (e.g., [27]). Beyond any doubt, ours were not the only studies demonstrating a possibility to reduce the toxicity of some metals with the help of this or that innocuous antagonist. For instance, it was shown that hepatic copper retention in ram lambs could be reduced by dietary supplementation with molybdenum and zinc [28]; or that quercetin, and especially quercetin in combination with arginine ameliorated nano-zinc oxide’s nephrotoxicity for rats [29]. Our own experiment demonstrated that both systemic toxicity and *in vivo* genotoxicity of silver nanoparticles were markedly attenuated against the background of oral administration of a multi-component bioprotective complex [7]. However, as far as we know, it remains to be the only example of successful bio-protection against any adverse effects of metal-containing nanoparticles. Thus, we thought it worthwhile to try and apply the same approach to copper oxide nanoparticles as well.

## 2. Results and Discussion

In this Section, we might have presented and discussed all the detected effects of the toxic action of copper oxide NPs first and then pass on to the preventative efficacy of the bio-protective complex tested. However, although the toxicity reducing effect of this complex is important primarily from the practical point of view, it is also a research tool that helped us to additionally verify each assumption in relation to the causality of this or that difference between the group exposed to the toxic impact and the control group which was not exposed to it, and to demonstrate that it is indeed due to this impact rather than to small sample errors. Despite the importance of the standard statistical methods for null hypothesis testing, they always leave room for doubt; meanwhile, there is a long established diagnostic principle in medicine known as ex juvantibus, which can be used in experimental toxicology as well. For these reasons we suggest discussing almost every toxic outcome in parallel with the discussion of changes in it brought about by bioprotectors.

### 2.1. Copper Accumulation in Different Organs and Morphological Changes Caused by it

After the period of repeated i.p. injections of copper-oxide nanoparticles, all investigated internal organs display more or less marked retention of copper judging by an increase in its concentration in comparison with the organs of rats from the control group, which was statistically significant in kidneys and liver (Table 1) Earlier, we [7] had found in an experiment of similar design involving equidimensional silver and gold nanoparticles that the accumulation of these metals in the organs differed depending explicitly on their comparative solubility: compared with silver, the less soluble gold was found in greater quantities in the liver and the spleen but in lesser quantities in the kidneys. In a similar experiment with exposure to Fe_3_О_4_ nanoparticles of diameter 10 or 50 nm, their retention in the liver and the spleen was lower in the first case, which was also associated with higher solubility due to greater specific surface area of the 10 nm particles [2,3].

From this perspective, the following is notable: while being highly stable in a suspension prepared in deionized water, copper oxide nanoparticles fully dissolved not only in 0.9% NaCl but also in a model biological medium, which in our experiment was supernatant obtained by centrifuging bronchoalveolar lavage fluid from unexposed rats. One and a half hours after being placed into such medium, they were not longer detectable as particles.

Thus, we believe that kidneys accumulate mostly the copper achieving this organ in ionic form after the nanoparticles’ *in vivo* dissolution, while organs rich in RES, such as liver and spleen are accumulating from blood (mainly by phagocytosis) predominantly nanoparticles which have not been completely dissolved as yet. In due time, nanoparticles retained in these organs are dissolved here and Cu-ions are being continuously released into bloodstream, but in conditions of repeated exposures new echelons of not dissolved nanoparticles are being captured by resident macrophages, *etc.* As to the brain, it is not as rich in phagocytes as liver or spleen and, although it is richly perfused, still less so as compared with kidneys. Indeed, even in humans the cerebral blood flow is usually estimated as *ca.* 750 mL/min (15% of the cardiac output) while the renal blood flow—as 1000–1100 mL/min (22% of the cardiac output). Taking into account significantly smaller brain-to-body mass ratio in rats as compared to humans, one may assume that percentage of total circulating blood volume flowing through the brain in these animals is even lower. We think these considerations can explain why copper content of brain is the lowest.

**Table 1:** Copper content of kidneys, liver, spleen and brain (mcg/g of dry-frozen tissue) of rats after repeat intraperitoneal injections of copper oxide nano-suspensions and/or oral administration of the bioprotective complex (BPC) (*х* ± *S*_х_).

Group of Rats Given	Kidneys	Liver	Spleen	Brain
**Water (control)**	42.4 ± 2.9	12.2 ± 2.4	22.5 ± 2.1	18.9 ± 0.7
**Nano copper oxide**	62.5 ± 7.1 *	28.8 ± 6.3 *	24.2 ± 1.5	21.5 ± 1.7
**Nano copper oxide + BPC**	59.4 ± 10.0	22.1 ± 3.5 *	18.0 ± 2.5 °	18.8 ± 1.4
**BPC**	50.4 ± 5.6	10.6 ± 0.3	25.3 ± 2.2	20.8 ± 1.5

Statistically significant (*p* < 0.05 by *t* Student’s test) differences from the control group are marked by *; those from the NP group by °.

We maintain, however, that both mechanisms (phagocytosis of particles and distribution of Cu-ions released from them) underlie copper accumulation in any organ of rats exposed to CuO-nanoparticles, and we can but hypothesize which of them prevails in this or that tissue.

One could assume that nanoparticle solubility plays a dual toxicokinetic role. On the one hand, it interferes with long-term persistence in any organ of NPs that first penetrated into the blood (from the abdominal cavity in this experiment, or from the lungs and gastro-untestinal tract in natural conditions), and then from the circulating blood into the cells of the organs into which they can penetrate not only by diffusion but also as a result of active endocytosis. Obviously, the latter mechanism prevails for the cells of reticulo-endothelial systems (RES) and for circulating or resident phagocytes. On the other hand, wherever nanoparticle dissolution is taking place (in the site of its primary deposition or in the organ to which it has been transported by blood), release of metal ions and their diffusion maintain a certain concentration of these ions in the blood—the so-called central toxicokinetic pool. Thus, the dissolution process causes both elimination of metal ions with urine and bile and its transfer to all tissues.

Unfortunately, unlike the experiment with iron oxide Fe_3_О_4_, the content of which, in just this chemical form (and, thus, in the form of magnetite particles), was measured experimentally by means of electron paramagnetic resonance (EPR) [2,3], our experiment with non-paramagnetic copper nano-oxide provided only data on the total content of this element in different organs (see the Experimental Section). In this case, therefore, we can judge about the relationship between the two toxicokinetic mechanisms under consideration only hypothetically, relying on some indirect evidence. Note, in particular, that the copper content of the spleen exposed to copper-oxide NPs was only slightly increased over the background level (by 8%) and was about the same for the brain (by 14%), whilst being 1.5 times higher than the background level for the kidneys and 2.4 times higher for the liver. Meanwhile, both liver and spleen abound in resident macrophages, and this logically explained why these two organs were comparably “greedy” (the spleen being even greedier) with regard to the ability to accumulate iron, silver and gold in experiments with corresponding nanoparticles. Thus, the fact that this comparability of metal retention in the spleen and liver was not observed in the experiment with copper oxide NPs provides indirect evidence against the key role of their endocytosis by the specialized cells and, consequently, in favor of the prevailing importance of Cu-ion distribution mechanisms. In this case, the difference between the organs in metal accumulation depends, most likely, on differences in perfusion (in terms of blood supply volume per unit mass of the organ), on the capillary network density (*i.e.*, on the total surface area of the diffusion membrane), on selective permeability of cell membranes for different ions, and on some other features of intracellular chemistry.

This suggests that the highly expressed accumulation of excess metal in the liver is in agreement with the key role of this organ in the maintenance of normal homeostasis of copper as an essential trace element [30]. Moreover, in the course of intoxication the excess copper arriving with the arterial blood into the liver, like in any other organ, is eliminated from the liver with bile and is then partially absorbed in the intestines and then gets back into the liver by the portal vein. This “vicious circle”, characteristic of not only copper toxicokinetics, is quite likely to additionally facilitate considerable accumulation of the metal in this organ.

**Figure 3:**
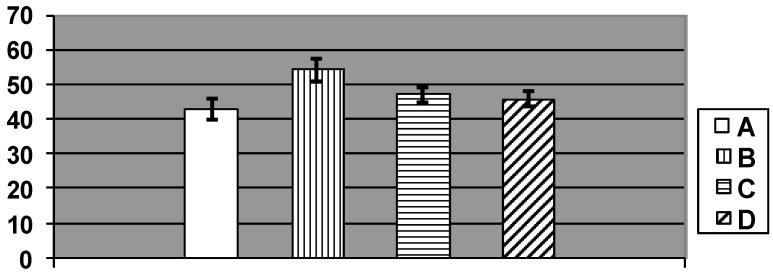
Number of Kupfer cells per 100 liver cells in rats exposed (A) to water (control); (B) to water suspension of copper oxide nanoparticles; (C) to the same against the background of BPC administrations; and (D) to the BPC only. (Average values with 95% CI). Differences are statistically significant between (B) and (A), and between (C) and (B) (*p* < 0.05 by Student’s *t*-test).

This reasoning might seem to be in contradiction with the fact that the number of Kupffer cells (*i.e.*, active resident microphages of the liver) at exposure to NPs was statistically significantly increased (Figure 3), as we observed it under subchronic exposure to iron oxide [2,3], silver, or gold [7] NPs. This, however, may be unrelated to the capture by Kupffer cells of any NPs from the blood, since it is well known that resident macrophages play multiple roles in the process of apoptosis of an organ’s functional cells, engulfing, in particular, the apoptotic bodies (e.g., [31]), and that in the liver just the Kupffer cells play this role [32]. Thus, Kupffer cell activation may serve as indirect indicator of copper’s cytotoxic effect, which is known to be associated with apoptosis, at least, for copper NPs [33]. The lack of this effect of NPs against the background of bio-protectors reducing copper toxicity as seen in the same chart (Figure 3) provides evidence in favor of such interpretation.

**Figure 4:**
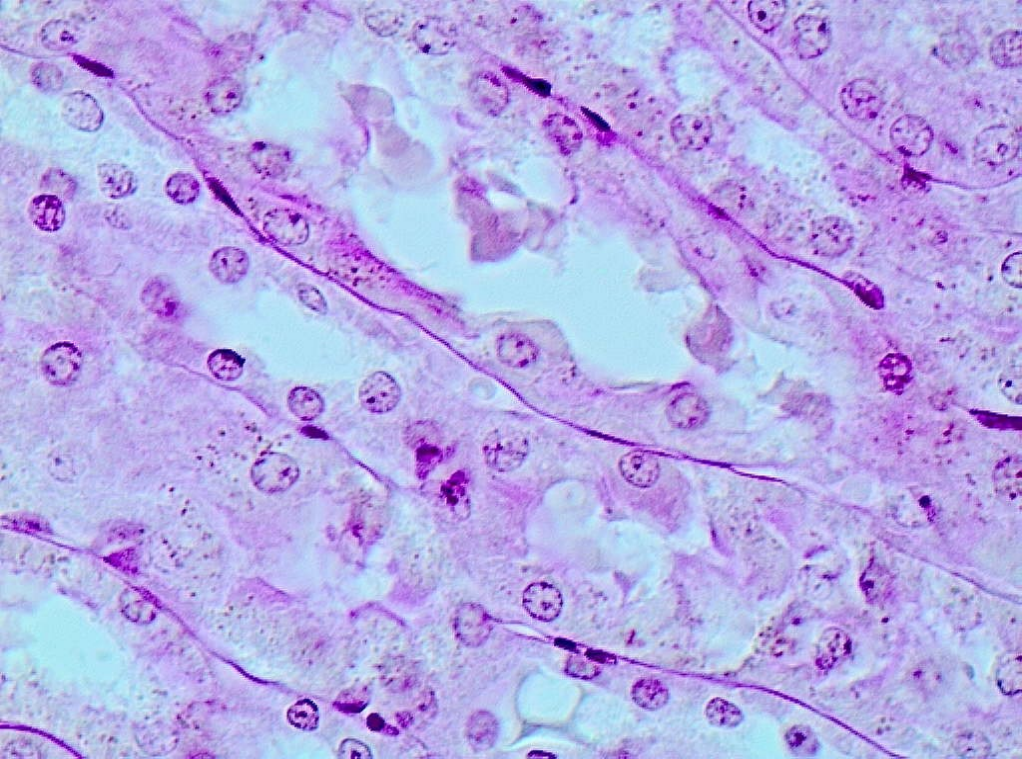
Kidney of a rat exposed to copper oxide nanoparticles. Marked degenerative and necrobiotic changes of tubular epithelial cells; partial destruction of the brush border; small granules of a pigment within сells and tubular lumen. PAS stain, magnification ×40.

Continuing to compare the retention of copper in the internal organs with histological signs of damage to them, it seems but logical to go over to kidneys, which rank second in the level of such retention. One may assume that considerable concentration of copper in the kidney is likely to be associated with just the peculiarities of its haemodynamics rather than with the excretory role of this organ, as it is known that the excretion of copper with urine is much less important than elimination with bile and further on with feces [29]. At the same time, as follows from the microphotograph (Figure 4) and morphometric data (Table 2), the kidneys,and tubular epithelial cells, in the first place, show obvious signs of toxic damage. This observation is consistent with the data obtained by Liao and Liu [18], who found that a five-day oral exposure to copper NPs at a dose of 50, 100 or 200 mg/kg, rather than microparticles at a dose of 200 mg/kg, caused extensive necrosis of cells in the proximal renal tubules. Xu *et al.* [24] showed in experiments “*in vitro*” that CuO nanoparticles cause oxidative stress leading to podocyte apoptosis.

**Table 2:** Some morphometric indices for the status of kidneys in rats after repeat intraperitoneal injections of copper oxide nano-suspensions and/or oral administration of the BPC (*х* ± *S*_х_).

Groups of Rats Given	Glomerular Surface Area (mm^2^)	Urinary Space (mm^2^)	Brush Border Loss (% Lengthwise)	Epithelial Desquamation (% Lengthwise)
**Water (control)**	2.47 ± 0.06	0.71 ± 0.04	5.39 ± 0.42	0.33 ± 0.13
**Nano copper oxide**	2.43 ± 0.04	0.89 ± 0.03 *	8.36 ± 0.76 *	1.16 ± 0.38 *
**Nano copper oxide + BPC**	2.82 ± 0.075 *	0.97 ± 0.05 *^,^▪	5.98 ± 0.46 ▪	0.98 ± 0.35
**BPC**	2.41 ± 0.05	0.74 ± 0.05	6.03 ± 0.57	0.73 ± 0.21

*, statistically significant difference from the control group; ▪, from the group given nano copper oxide (without the BPC); *p* < 0.05 (*р*, values are Bonferroni-corrected for multiple comparisons).

These manifestations of nephrotoxicity were considerably attenuated in the case of exposure to NPs against the bioprotective complex (BPC) administration, which is unlikely to be explained by a small (by 5% only) decrease in the accumulation of copper in the kidneys and is more likely to be associated with an increase in the resistance of the organism to its toxic action. The reasons for the increase in the urinary space will be touched upon below in connection with increased diuresis in the rats administered the BPC.

Although the increased copper content of the spleen and brain was statistically insufficiently significant, the unidirectionality of this shift in all the four organs investigated renders its accidental character hardly probable.

**Figure 5:**
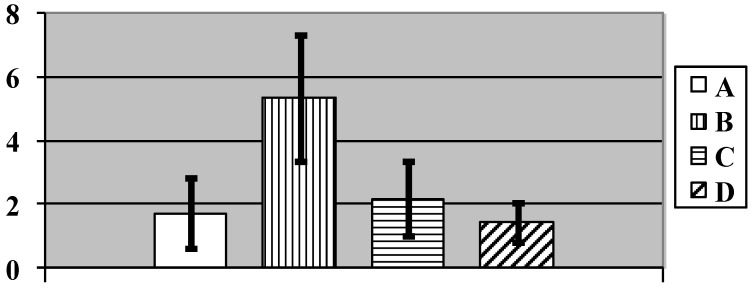
Number of brown pigment micro aggregates per square of Avtandilov’s grid in the spleen’s red pulp of rats exposed (A) to water (Control); (B) to water suspension of copper oxide nanoparticles; (C) to the same against the background of BPC administrations; and (D) to the BPC only. (Average values with 95% CI). Differences are statistically significant between (B) or (C) *vs.* (A), and between (C) *vs.* (B) (*p* < 0.05 by Student’s *t*-test).

**Figure 6:**
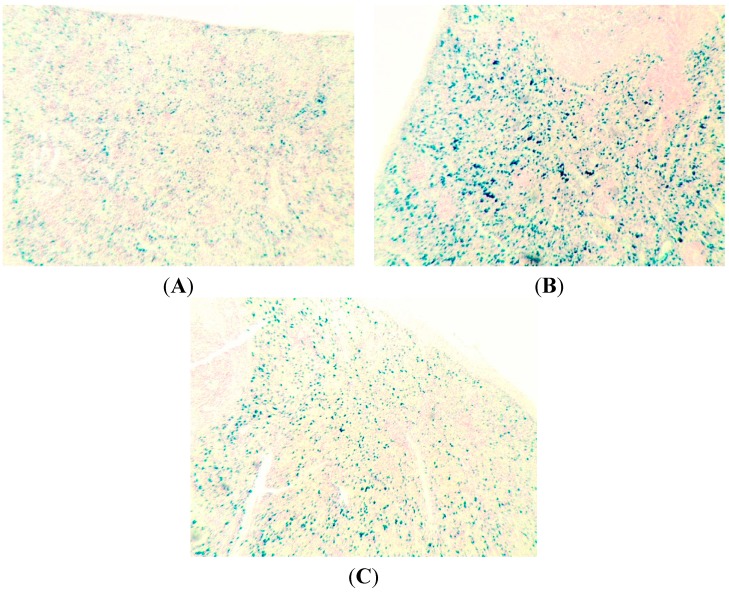
Hemosiderin distribution the spleen’s red pulp of rats exposed (**A**) to water (Control); (**B**) to water suspension of copper oxide nanoparticles;and (**C**) to the same against the background of BPC administrations. Perl’s stain; magnification ×60.

The main finding from the examination of the spleen structure is an increase in brown pigment granules in the red pulp in both the macrophage cytoplasm and extracellularly. This phenomenon was estimated by average total number of pigment agglomerates within Avtandilov’s grid with the following results: the average index was equal to 1.72 ± 0.34 in the control group and 1.43 ± 0.18 in the group receiving only BPC, whereas in the “NPs only” group it was 5.33 ± 0.61, and in the NPs plus BPC one it was 2.18 ± 0.36 (the difference of the latter two groups between them and between each of them and the control group is statistically significant at *p* < 0.001 by Student’s *t*-test). This result, being in good agreement with other data confirming the protective effect of BPC, is illustrated by the chart in Figure 5. Insofar as the additional Perl’s stain of spleen sections for iron (Figure 6) provide evidence in favor of this pigment being hemosiderin, then it would be logical to relate both its accumulation in the spleen in response to nano-copper and the decrease of this effect of nano-copper against the BPC administration to intracellular haemolysis and its attenuation, respectively (see Table 4 below).

Of greatest toxicological importance is the increased (by 14%) copper content of the brain, considering the special role of such accumulation in certain genetically caused disturbances in the kinetics of copper in the organism [34]. A histological examination of the brains of the rats exposed to NPs revealed pathological changes of neurons in the ganglion cell layer of the cortex and in basal nuclei. These changes are of special interest, because it is damage to the basal ganglia and frontal cortex, and neuronal apoptosis [35] in particular, that is associated with the neurotoxic action of free copper in Wilson’s disease. Interestingly, we revealed some laboratory signs typical of this disease (namely, increase in the copper content of the liver and brain against a lowered level of ceruloplasmin in the blood serum) in response to subchronic exposure of rats to copper-containing nanoparticles (see Table 1 and Table 4).

**Figure 7:**
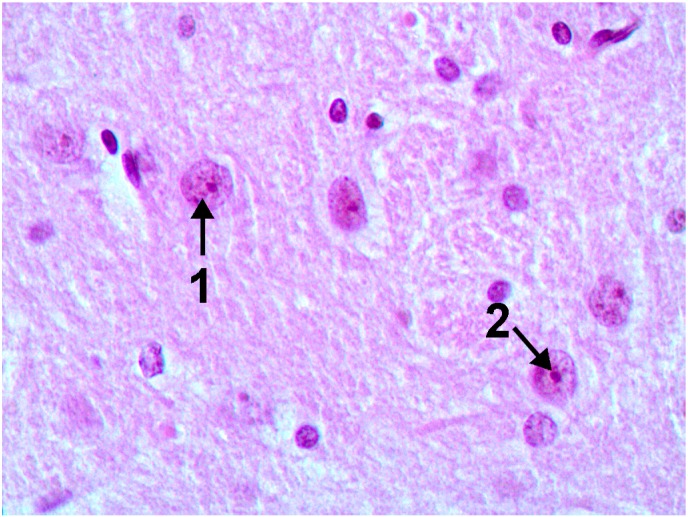
The brain of a control rat, the nucleus caudatus area (hematoxylin-eosine stain, magnification ×400). The neuron nuclei are predominantly spherical with well-visible eosinophilic granulosity (1), and notable nucleoli in the center (2).

**Figure 8:**
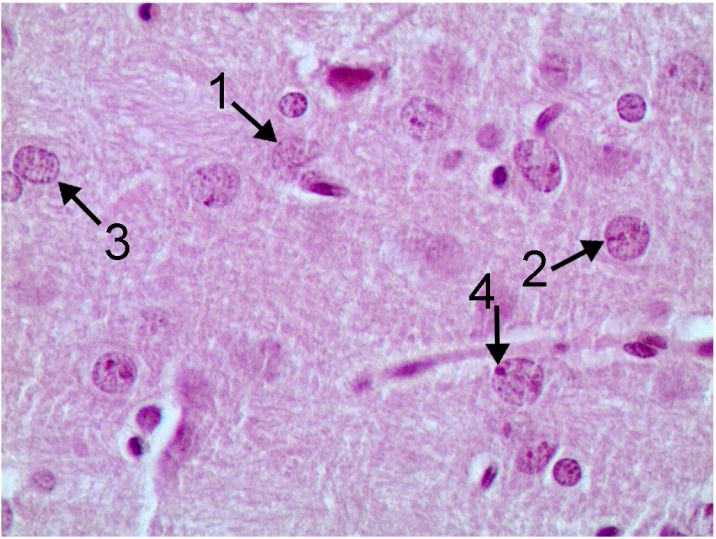
The brain of a rat exposed to subchronic intoxication with copper oxide nanoparticles, the nucleus caudatus area (hematoxylin-eosine stain, magnification ×400). Neuron nuclei stain poorly, with an indistinct membrane (1), the nucleoli are pycnotic (2), are often absent (3) or are shifted towards the nuclear membrane (4).

It is especially interesting that a histological examination of one of the basal nuclei, namely nucleus caudatus (for identifying which the rats’ brain sections have reliable anatomical landmarks), revealed clear signs of damage to its neurons—Golgi cells (compare Figure 7 and Figure 8). In these cells, we observed poor staining of the eosinophilic granulosity of nuclei, disappearance of the nuclear membrane, and pycnosis of the nucleolus, which is often displaced towards periphery of nucleus or is absent. Whether or not all of these features may be interpreted as light microscopy signs of neuronal apoptosis or of other types of neuron’s toxic damage is difficult to decide without special tests (e.g., an immuno-histochemical detection of caspase 3) but we think that literature data concerning apoptosis of neurons in Wilson’s disease [35] are rather suggestible.

**Figure 9:**
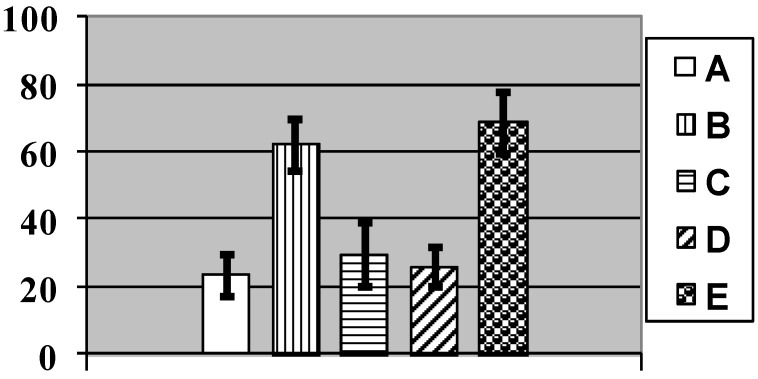
Number of cells without a nucleolus per 100 Golgi cells in nucleus caudatus of rats exposed (A) to water (Control); (B) to water suspension of copper oxide nanoparticles; (C) to the same against the background of bioprotective complex (BPC) administrations; (D) to the BPC only; (E) to water suspension of Cu/Cu_2_O submicron particles (Average values with 95% CI). Differences are statistically significant between (B) and (A), (E) and (A), and (C) and (B) (*p* < 0.05 by Student’s *t*-test).

For morphometry, we used absence of the nucleolus as the most accurate and unbiased index. As follows from the chart (Figure 9), in control rats the average number of such cells without nucleoli per 100 Golgi cells count was 23.3 ± 2.8, while in rats exposed to NPs it was 62.2 ± 3.1. That this is an effect of intoxication is confirmed not only by its statistical significance (at *p* < 0.05), but also by the fact that it was also observed in a parallel experiment with the same doses of submicron copper particles: 68.7 ± 3.9. (The effect on the organism of specially prepared particles having an average diameter of 340 ± 168 nm (*x* ± s.d.), a copper suboxide surface layer of about 80 nm in thickness with an atomic ratio of Cu:O = 2:1 and a metallic copper core was estimated in an experiment of similar design carried out at the same time using the same indices as the ones used in this paper to characterize the impact of copper-oxide NPs That impact is the subject matter of a special publication in preparation, but we believe it is worthwhile mentioning it in this context since it confirms the dependence of the described damage to basal nuclei on copper toxicity. Again, exposure to copper oxide NPs with the administration of the BPC provided only a small and statistically insignificant increase in the index considered (29.5 ± 4.14); the latter did not differ also from the controls for the administration of BPC without nanoparticles (NPs) (26.1 ± 2.59).

**Figure 10:**
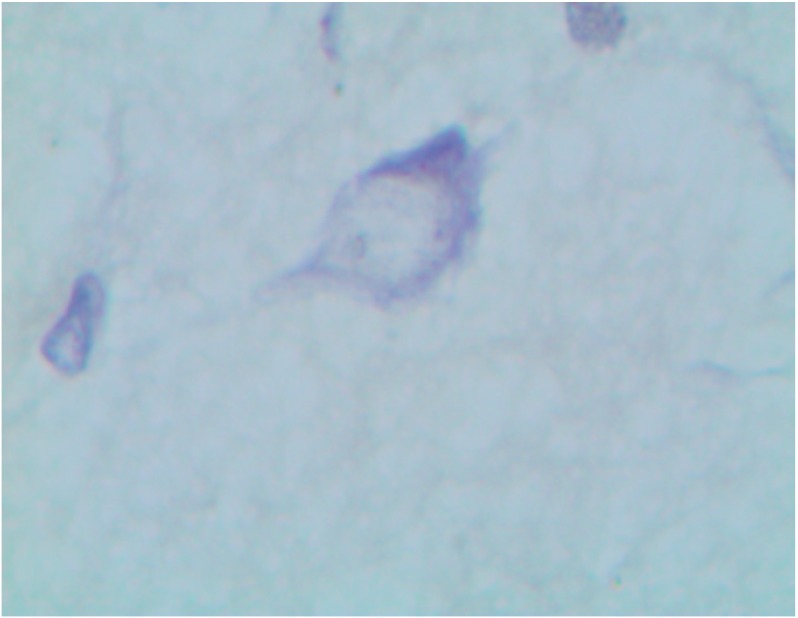
The brain of a control rat, the nucleus caudatus area (Nissl stain, magnification ×900). A typical neuron with evenly spread tygroid in the soma and the axon hillock.

**Figure 11:**
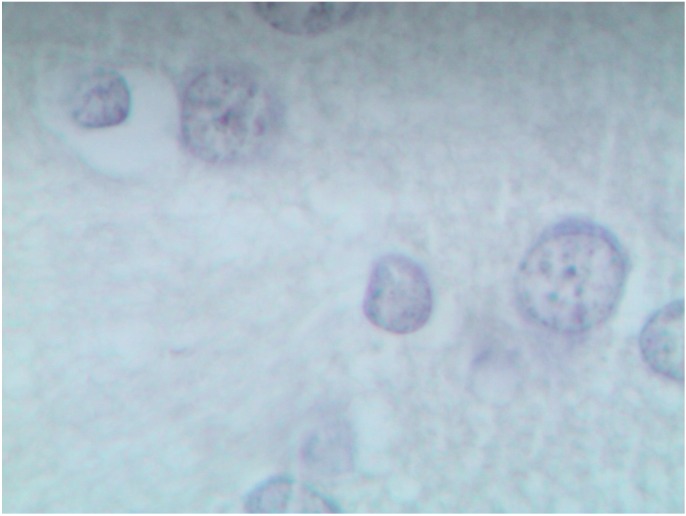
The brain of a rat exposed to subchronic intoxication with copper oxide nanoparticles, the nucleus caudatus area (Nissl stain, magnification ×900). A total or subtotal loss of tigroid.

**Figure 12:**
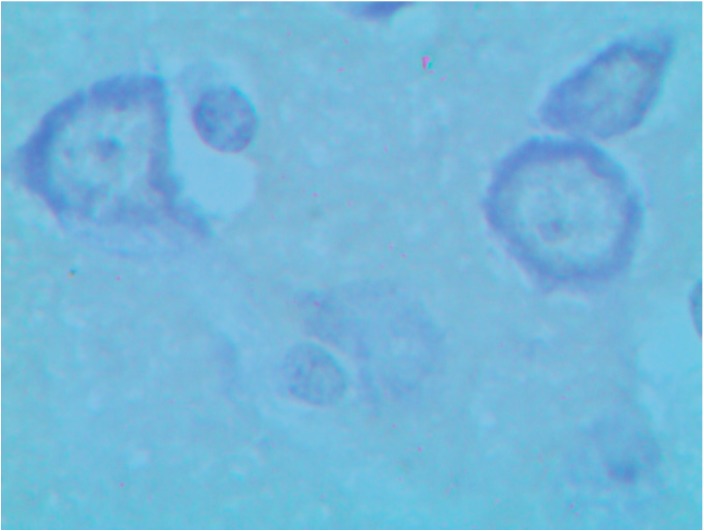
The brain of a rat exposed to subchronic intoxication with copper oxide nanoparticles against the background of bioprotective complex administration, the nucleus caudatus area (Nissl stain, magnification ×900). The tigroid’s distribution is similar to that in control rats (compare with Figure 10).

Nissl staining gave additional information confirming damage to neurons of basal nuclei induced by copper oxide NPs intoxication and attenuation of this damage by the BPC (see Figure 10, Figure 11 and Figure 12).

The genotoxicity of copper-containing nanoparticles, established by a number of researchers mainly in experiments *in vitro* (see Background Section), manifested itself in our experiment with subchronic exposure of rats to copper oxide NPs in enhanced DNA fragmentation in liver and spleen cells (Table 3). The fact that there was no increase in the coefficient of nuclear (genomic) DNA fragmentation in the brain cells is most likely to be explained by the absence of mitotic activity in neurons rather than by low accumulation of copper in this tissue. Indeed, this increase was observed in spleen cells, in which the copper content was even less elevated compared with the controls. Meanwhile, the disappearance of the nuclear membrane in the course of mitosis, starting with the prometaphase and including the meta = and anaphases, renders the genomic DNA more accessible for contact with any damaging factor that has penetrated into the cell. Therefore we may assume that non-proliferating cells withstand genotoxic action better as compared with proliferating ones.

**Table 3:** Coefficients of the genomic DNA fragmentation in rats after repeat intraperitoneal injections of copper oxide nano-suspensions and/or oral administration of the bioprotective complex (BPC) based on the results of Random Amplification of Polymorphic DNA (RAPD)-test, (*х* ± *S*_х_).

Organs	Groups of Rats Given			
	Nano Copper Oxide	Nano Copper Oxide + BPC	BPC	Water (Control)
**Liver**	0.426 ± 0.0020 *	0.404 ± 0.002 *^,^▫^,^▪	0.394 ± 0.0040	0.396 ± 0.0020
**Kidneys**	0.382 ± 0.0015	0.406 ± 0.0027 *^,^▫^,^▪	0.393 ± 0.0025 *	0.383 ± 0.0025
**Spleen**	0.460 ± 0.0020 *	0.418 ± 0.0015 *^,^▫^,^▪	0.377 ± 0.0028 *	0.369 ± 0.0016
**Brain**	0.355 ± 0.0020	0.335 ± 0.0021 *^,^▫	0.356 ± 0.0025	0.354 ± 0.0028

*, statistically significant difference from the control group; ▫, from the group given the BPC only; ▪, from the group given nano copper oxide (without the BPC); *p* < 0.05 (Student’s *t*-test).

The absence of any visible damaging action of the intoxication on renal DNA could be explained only by the fact that the tubular epithelial cells suffered most from the cytotoxic action of copper (up to full desquamation), taking into consideration that cell death is an alternative to cell mutation. This hypothesis is indirectly supported by quasi-paradoxical detection of a genotoxic effect of copper oxide NPs where just the nephrotoxic effect of copper is attenuated by the action of bioprotectors. On the contrary, the genotoxicity of copper in liver and spleen cells, although explicit even against the background of BPC administration, was still significantly attenuated by it.

It is important to emphasize that we revealed the genotoxic effect of copper-containing nanoparticles “*in vivo*” after a subchronic exposure that caused just moderate signs of intoxication judging from the set of functional and biochemical indices (see below). This fact permits to suggest, tentatively, that the genotoxicity (and presumably carcinogenicity) may be one of the main hazards associated with these NPs.

In the foregoing discussion of the effects of BPC on outcomes of copper accumulation in different organs we virtually skipped its effect on this accumulation itself. Nevertheless, after exposure to copper-oxide NPs with the administration of BPC, the copper content of all organs examined was always lower than without BPC administration (Table 1). This reduction was statistically significant only for the spleen, but the unidirectionality of inter-group differences for all the four organs is unlikely to be accidental. The simplest explanation of the decrease in the organ’s copper burden due to BPC is that the enterosorbent contained in this complex (namely, pectin) prevents partial re-absorption of this metal into the blood from the intestines where the bile delivers it to. However, in an earlier experiment of similar design with silver nanoparticles [7], we also found a whole range of positive effects from a BPC that was essentially similar though not identical in composition, but we not discover any effect of that BPC on silver accumulation in the organs. Given the fact that the liver and intestines are the main organs for elimination of, not only copper, but also silver, we cannot yet explain this difference between the results of our two experiments.

### 2.2. Functional and Biochemical Signs of Intoxication

As can be seen from Table 4, judging by the shifts in the functional and biochemical indices of the organism’s status, the subchronic toxic effects of copper-oxide NPs in the used dosage appear to be moderate enough. Nevertheless, the exposure to them has caused:

- A statistically significant increase in mass coefficients of the liver, kidneys and spleen;
- Statistically significant adverse changes in the red blood cells (a decrease in the haemoglobin content and number of erythrocytes with an increased percentage of reticulocytes—this result is most likely due to the haemolytic effect of copper) and in the porphyrin metabolism (an insignificant increase in renal excretion of coproporphyrin with a statistically significantly reduced excretion of delta-aminolevulinic acid);
- General inhibition of oxidative metabolism (judging by the statistically significant decrease in the SDH activity of blood lymphocytes), and of lipid peroxidation (decreased renal excretion of malonyldialdehyde (MDA)).

It should be especially noted that exposure to NPs caused an appreciable (though statistically not significant) decrease in the levels of ceruloplasmin and both aminotransferases (alanine- and asparate-transaminases (ALT, AST)) in blood serum. Traditionally, it is the release of these and some other enzymes produced by the liver into the blood serum that is considered as a sign of liver disease or toxic damage. Indeed, Pal *et al.* [36] discovered an increase in the level of ceruloplasmin after a 90-day exposure of rats to copper, which should be considered just as a sign of copper hepatotoxicity since ceruloplasmin is not copper-containing transport protein. However, there is evidence that the release of hepatic enzymes, for example ALT, is preceded by a phase of decrease in its blood level, which can be explained by either the suppression of its synthesis by hepatocytes or the inhibiting effect of the toxic metal on its activity. Thus, for instance, upon a single i.p. injection of nano-silver Lee *et al.* [37] observed a decrease in blood serum ALT in comparison with a parallel control in 1 and 4 days, absence of difference with the controls in 7 and 10 days, and subsequent increase only in 30 days.

A favorable difference of the organism’s status in the group receiving NPs plus BPC compared with the group receiving NPs without BPC is notable by a number of indices, in spite of the fact that harmful toxicodynamic effects of nanoparticles even without bio-protection were moderate or even weak (in such cases it is usually difficult to detect their attenuation by any bioprotectors). In particular, BPC administration was accompanied by a reduction or even disappearance of such NP-related shifts (even if statistically insignificant in the “NP only” group) as:

- A reduced hemoglobin content against an increased percentage of reticulocytes;
- A lowered protein-producing function of the liver (judging by total protein and albumin contents of the blood serum);
- A lowered level of oxidative metabolism (judging by the succinate dehydrogenase activity of blood lymphocytes) and of lipid peroxidation (judging by the MDA content of the blood serum).

At the same time, the rats receiving copper oxide NPs and BPC did not display any statistically significant adverse differences from the group receiving the same NPs without BPC. As to the BPC alone (without NPs), its administration caused statistically significant shifts compared with the controls only for two indices out of 39: Increased kidney mass coefficient paralleled by statistically insignificant but notable increase in diuresis and increased activity of alkaline phosphatase in blood serum. The increased kidney mass is likely to be a sign of increased functional activity of the organ (including enhanced blood supply), resulting just in increased diuresis (also marked in the NPs + BPC group), as well as related increased urinary space revealed by morphometry of histological kidney preparations (see Table 2). All this is consistently explained by the fact we noticed long ago: rats usually drank sodium glutamate water solution (included into the BPC tested by us) in a much greater volume than drinking water given also ad lib to rats in the control group.

Given the absence of any morphological or functional signs of damage to the liver in the group receiving BPC alone, it would be stretch to interpret the increase in the release of alkaline phosphatase into the blood as a sign of such damage. We believe it admissible to regard the increased activity of this important enzyme (controlling the dephosphorylation process) rather as a positive effect of bioprotectors although we find it difficult to suggest any mechanisms of this beneficial action.

On the whole, we maintain that we demonstrated the positive efficacy of the tested biopreventive complex.

**Table 4:** Indices for the status of the organism of rats after repeat intraperitoneal injections of copper oxide nano-suspensions and/or oral administration of the bioprotective complex (BPC) (*х* ± *S*_х_).

Indices	Groups of Rat Receiving				
	Nano Copper Oxide	Nano Copper Oxide + BPC	BPC	Water (Controls)	
Initial body mass, g	178.8 ± 2.5	175.8 ± 1.7	178.8 ± 4.9	174.6 ± 2.7	
Body mass after period of injections, g	225.8 ± 8.0	232.5 ± 6.2	227.5 ± 6.8	217.0 ± 6.4	
Temporal summation of sub-threshold impulses, s	12.2±1.1	12.35 ± 1.6	13.4 ± 0.9	13.4 ± 0.8	
Number of head-dips into holes during 3 min	6.1± 1.1	5.1 ± 1.0 *	8.1 ± 1.0	9.1 ± 1.70	
Haemoglobin, g/L	13.4 ± 0.4 *	14.3 ± 0.3 *^,^▪^,^▫	16.7 ± 0.5	16.6 ± 0.4	
Erythrocytes, 10^12^ g/L	1.6 ± 0.1 *	1.63 ± 0.0 *	1.69 ± 0.1	1.84 ± 0.0	
Reticulocytes, ‰	28.9 ± 2.1 *	16.0 ± 1.6 *^,^▪^,^▫	11.0 ± 1.9	10.45 ± 0.8	
Lymphocytes, %	56.9 ± 2.9	52.3 ± 3.4	55.2 ± 3.1	56.3 ± 2.2	
Segmented neutrophils, %	57.7 ± 4.2	62.9 ± 5.3	60.7 ± 4.3	54.7 ± 2.1	
Band neutrophils,%	3.2 ± 0.7	3.9 ± 0.7	3.6 ± 0.5	3.1 ± 0.8	
Monocytes, %	19.6 ± 2.2	19.6 ± 2.8	16.6 ± 2.3	18.4 ± 1.4	
Eosinophils, %	7.0 ± 1.4	8.0 ± 1.5	7.3 ± 2.2	10.3 ± 1.4	
Basophils, %	0.6 ± 0.2	1.1 ± 0.3	0.73 ± 0.2	0.9 ± 0.3	
Total protein in blood serum, g/L	70.3 ± 1.2	74.8 ± 0.7 ▪	73.2 ± 1.4	72.4 ± 1.3	
Albumins in blood serum, g/L	36.9 ± 0.7	40.6 ± 0.6 ▪	39.1 ± 0.5	38.3 ± 1.0	
Globulins in blood serum, g/L	33.3 ± 1.0	34.2 ± 0.7	34.1 ±1.2	34.0 ± 1.1	
A/G index	1.1 ± 0.0	1.2 ± 0.0	1.16 ± 0.0	1.14 ± 0.1	
SDH activity, number of formasan granules in 50 lymphocytes	656.8 ± 12.0 *	807.5 ± 20.9 *^,^▪	766.1 ± 19.5	737.1 ± 10.7	
SH-gtoups, mmol/L	1.1 ± 0.0	1.0 ± 0.1	1.1 ± 0.0	1.1 ± 0.0	
GSH-gtoups, mmol/L	0.6 ± 0.1	0.5 ± 0.1	0.4 ± 0.1	0.5 ± 0.0	
ALT activity in blood serum, mmol/h L	49.3 ± 2.7	54.6 ± 3.3	62.5 ± 3.9	52.8 ± 3.6	
AST activity in blood serum, mmol/h L	234.8 ± 13.3	218.8 ± 14.5	235.9 ± 16.5	254.7 ± 16.8	
Catalase in blood serum, μmol/L	1.4 ± 1.1	1.2 ± 0.2	1.5 ± 0.1	1.2 ± 0.2	
MDA in blood serum, nmol/L	3.7 ± 0.2 *	4.3 ± 0.2 ▪	4.1 ± 0.2	4.0 ± 0.1	
Ceruloplasmin in blood serum, mg %	80.0 ± 5.5	100.5 ± 3.1 ▪	109.9 ± 8.0	90.9 ± 45.0	
Bilirubin in blood serum, μmol/L	2.4 ± 0.3	2.8 ± 0.4	3.2 ± 0.6	2.8 ± 0.6	
Alkaline phosphatase in blood serum, nmol/s L	117.5 ± 11.1	185.7 ± 18.5 *^,^▪	142.8 ± 11.4 *	97.8 ± 11.7	
γ-Glutamintransferase, units/L	3.5± 0.8	4.1 ± 1.1	5.6± 1.4	3.7 ± 1.0	
Creatinine in blood serum, μmol/L	34.6 ± 1.34	35.2 ± 1.43	35.8 ± 1.3	36.8 ± 1.0	
Daily volume of urine, mL	39.9 ± 6.2	42.6 ± 4.8	43.9 ± 6.3	38.3 ± 7.0	
Creatinine in urine, mol/L	1.1 ± 0.3	0.8 ± 0.1	0.8 ± 0.1	0.8 ± 0.1	
Coproporphyrin in urine, nM/L	89.2 ± 9.5	76.4 ± 13.4	85.2 ± 17.2	69.0 ± 12.1	
δ—ALK in urine, μmol/L	5.9 ± 1.1 *	6.3 ± 1.3	8.3 ± 1.3	10.4 ± 1.8	
Liver mass, g per 100 g body mass	4.0 ± 0.1 *	4.0 ± 0.2	3.5 ± 0.1	3.6 ± 0.1	
Kidney mass, g per 100 g body mass	0.6 ± 0.0 *	0.7 ± 0.0 *	0.7± 0.0 *	0.4 ± 0.0	
Spleen mass, g per 100 g body mass	0.5 ± 0.0 *	0.5 ± 0.1	0.4 ± 0.0	0.4 ± 0.0	
Brain mass, g per 100 g body mass	0.8 ± 0.0	0.7± 0.0	0.8 ± 0.0	0.8 ± 0.0	

*, statistically significant difference from the control group; ▪, from the group given nano copper oxide (without the BPC); ▫, from the group given the BPC only; *p* < 0.05 (*р*, values are Bonferroni-corrected for multiple comparisons).

## 3. Experimental Section

The animal experiment was carried out on outbred white female rats from our own breeding colony with the initial body weight of 150 to 220 g, with minimum 12 animals in different exposed and control groups. Rats were housed in conventional conditions, breathed unfiltered air, and were fed standard balanced food. The experiments were planned and implemented in accordance with the “International guiding principles for biomedical research involving animals” developed by the Council for International Organizations of Medical Sciences (1985) and approved by the Ethics Committee of the Ekaterinburg Medical Research Center Medical for Prophylaxis and Health Protection in Industrial Workers.

For this animal experiment, we prepared a stable suspension of copper oxide nanoparticles by the method of laser ablation with the following increase in concentration by partial evaporation proved necessary to enable the administration of effective doses to rats in minimal volumes of water. A plate of copper with a metal content of 99.99% was placed on the bottom of a dish with deionized water. Metal ablation was performed using Fmark-20RL laser material processing system (by Laser Technology Center, Ekaterinburg, Russia), based on ytterbium-doped pulsed fiber laser (pulse length 100 ns, repetition rate 21 kHz, wavelength 1064 nm). The energy density was 80 J/cm^2^. The target was irradiated in scanning mode with a rate of the laser ray 270 mm/s (first 7 cycles of such scanning served to prepare the target’s surface).

**Figure 13:**
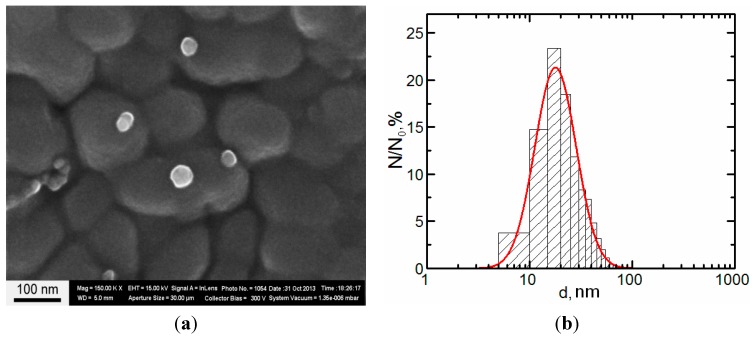
(**а**) Scanning electron microscopy (SEM) images of nanoparticles prepared for the experiment; and (**b**) particle size distribution function obtained by analysis of SEM images (N—number of particles of a given diameter, N_0_—total number of particles).

The concentration of the primary suspensions obtained by ablation was 0.08 mg/mL. An increase in concentration to 0.5 mg/mL was achieved by drying the suspensions for 5 h at 50 °C, which was not accompanied by nanoparticle aggregation. Particle images were obtained after concentrating by scanning electron microscopy (SEM) with the AURIGA CrossBeam Workstation (Carl Zeiss, Germany), which enabled us to identify their spherical form (Figure 13). The average particle diameter (±SD) obtained through statistical processing of hundreds of SEM images was 20 ± 10 nm, the distribution being symmetrical.

No essential changes took place 30 days after the preparation of the suspension in either zeta potential or the form and position of the plasmon resonance peak, providing evidence of its high stability. For studying the kinetics of NPs dissolution in a ionic medium or in model liquid biological medium we measured change in optical absorption at 620 nm wave length corresponding to maximum optical absorption of copper nanoparticles. Before measurement, the suspension of copper oxide nanoparticles with a concentration of 0.5 mg/mL was diluted fourfold by normal saline or supernatant of fluid obtained through bronchoalveolar lavage (BALF) of intact rats. Absorption spectra were measured by means of an UV-1650 spectrophotometer. Before each measurement, the solution was sonicated. We found that nanoparticles would completely vanish (presumably dissolving) within 20 min after the addition of normal saline and within 90 min after the addition of BALF.

The chemical composition of the nanoparticles was determined by X-ray energy dispersion analysis using an Auriga CrossBeam scanning electronic microscope (Carl Zeiss, Germany) equipped with an X-Max X-ray detector (Oxford, UK). Analysis of the characteristic X-ray radiation resulting from the bombardment of a target surface with a beam of accelerated electrons makes it possible to determine the chemical (element) composition in the area of interaction between electrons and the substance to a depth of several microns. Measurements were performed with an accelerating voltage of 5 kV and averaged over an area of 100 × 100 µm. The composition of nanoparticles (in terms number of atoms) was found to be equal to 53% ± 5% Cu and 47% ± 5%, *i.e.*, actually to 1:1, which corresponds to the chemical composition of nanoparticles in workroom air during the casting of refined copper (see Background Section) and close to CuO. However, it should be noted in this case, too, that they consist not only of СuO, since it is characteristic of nanoparticles formed during vapor condensation and generally for condensed phases to display substantial deviations from stoichiometry. Most likely, these nanoparticles consist of a mix of various copper oxides, which are impossible to identify individually.

The suspension thus prepared was administered to rats intraperitoneally 3 times a week (up to 19 injections) at a dose of 10 mg/kg (*i.e.*, about 2 mg per rat) in the corresponding volume of the suspension containing 0.5 mg of nanoparticles per mL.

It seems that using intraperitoneal injections for modeling systemic intoxication, which in real conditions that prompted us to conduct this experiment can be induced by inhalation exposure of workers (see Introduction) needs some justification. It is well known that “nanoparticles deposit with high efficiency in the entire respiratory tract, from the head airways to the alveoli, due to diffusion” [38]. For instance, the widely recognized Human Respiratory Tract Model (HRTM) of the International Commission of Radiological Protection (ICRP) [39,40] predicts 100% total deposition of 0.001 μm (*i.e.*, 1 nm) and ~90% for 0.01 μm (*i.e.*, 10 nm) particles (mostly in nose, pharynx, mouth, and larynx) for a normal adult mouth breathing male human subject. However there are many anatomical, functional and aerodynamic differences between humans and rodents which make one assume interspecies distinctions in regional particle deposition and thus in kinetics of their elimination to the GIT and/or absorption. It is no wonder that the authors of a recently published comprehensive review of nano-toxicological assessment techniques [41] maintain that “rodents, the commonly used species for toxicology testing, are obligatory nose breathers and, therefore, not representative models for human respiratory inhalation exposure”. In other words, nanoparticles inhalation by laboratory rodents is not as ideal a model of reality as it is often deemed to be.

The intraperitoneal model permits to circumvent these interspecies differences and is adequate enough when one looks for organ distribution and elimination of, and for organism’s reactions to nanoparticles after they penetrated into blood from an artificially created deposit. Like any experimental model (always a necessary simplification of a complicated system deliberately omitting some sub-systems and some material or informational flows and feedbacks) it has both drawbacks and virtues. Among the latter, one should take into consideration that dosing by injection is much more accurate and reliable as compared with the more “natural” experimental methods. This reason is crucial for experiments of comparative design like ours. Intraperitoneal modeling of subchronic intoxications is well known and recognized in general experimental toxicology. Moreover, it was used just in experimental nano-toxicological studies published in reputable journals (for instance, [2,3,7,42,43]).

As to the actual dose, it was chosen based on our experience as one inducing actual effects of subchronic toxicity (which might be attenuated at the background of bioprotectors but causing no deaths within the exposure period).

Animals in the control groups were injected with sterile deionized water (from the batch used for preparing suspensions) by the same route. An additional group of rats studied in parallel was being injected with the same dosage of NPs but against the background of administration of a bio-protective complex (BPC) described below, and still another group was given the same BPC plus i.p. injections of water.

Immediately after the end of the exposure period, the following procedures were performed for all rats:

- Weighing;
- Estimation of the CNS ability to the temporal summation of sub-threshold impulses—A variant of withdrawal reflex and its facilitation by repeated electrical stimulations in intact, conscious rat [44];
- Recording of the number of head-dips into the holes of a hole-board, which is frequently used for studying behavioral effects of toxicants and drugs (e.g., [45,46]);
- Collection of daily urine for analysis of its density, urine output, coproporhyrin, δ-aminolevulinic acid (δ-ALA), and creatinine contents;

Sampling of capillary blood from a notch on the tail for examining the haemogram, hemoglobin content, and for cytochemical determination of succinate dehydrogenase (SDH) activity in lymphocytes (by the reduction of nitrotetrazolium violet to formasan, the number of granules of which in a cell is counted under immersion microscopy).

Then the rats were killed by decapitation and blood was collected by exsanguination. The liver, spleen, kidneys, and brain were weighed. The biochemical indices determined from the blood included reduced glutathione (GSH), total serum protein, albumin, globulin, bilirubin, ceruloplasmin, malonyldialdehyde (MDA), alkaline phosphatase, alanine- and asparate-transaminases (ALT, AST), catalase, gamma glutamyl transferase, SH-groups, and creatinine. All the above-mentioned clinical laboratory tests on blood and urine were performed using well-known techniques described in many manuals (e.g., [47]).

The copper content of the liver, spleen, kidneys, and brain was determined by atomic emission spectrometer with inductively coupled plasma iCAP-6500 Duo (Thermo Scientific, Waltham, MA, USA). Samples of freeze-dried homogenized tissue were subjected to acid digestion with the help of a MARS 5 microwave accelerated reaction system.

Liver, spleen, kidney, and brain tissue sections were prepared from 4 rats in each treatment and control group for histological examination by haematoxilin-eosine stain and, where necessary, PAS, Nissl or Perl’s stain. We used the Avtandilov’s planimetric ocular grid for morphometric characterization of spleen (Figure 5) and image recognition programmed system CellSens (Olympus, Ekaterinburg, Russia) for that of kidneys (Table 2).

### 3.1. Testing of Genotoxicity (the Random Amplification of Polymorphic DNA (RAPD) Test, Random Amplification of Polymorphic DNA)

Totally, we analyzed 100 samples of liver, spleen, kidney, and brain, each sample in three replications. The organs were extracted from the bodies of the rats within 2–3 min upon killing into special vessels cooled to −80 °С. These were then promptly delivered in cryocontainers to a specialized laboratory, where they were minced with a scalpel and then subjected four times to freezing in liquid nitrogen and to thawing in an ultrasonic bath (38 °С) in PBS solution (Ca^2+^, Mg^2+^ free (Sigma-Aldrich, St. Louis, MO, USA) with subsequent passing through needles of decreasing diameter. To isolate DNA from the cells, we used a GenElute (Sigma) set of reagents in accordance with the manufacturer’s guidelines for use. The DNA content of the samples was determined spectrophotometrically (Ultraspec 1100 pro, Amersham Biosciences, Ltd., Amersham, UK), then they were freezed and stored at −84 °С in a kelvinator (Sanyo Electric Co., Ltd., Moriguchi, Japan) till the beginning of the implementation of the RAPD test. It was performed as described by us earlier [48,49]. This technique allows one to define quantitatively the degree of DNA fragmentation as an estimate of the genotoxicity of harmful agents and the protective effects of the set of bioprotectors studied. The method is based on the fact that, unlike a fragmented DNA, which forms the so-called “comet tail” in the agarose gel in electrophoresis, a non-fragmented DNA has a very low degree of migration and virtually stays in the same place (“comet head”), the degree of migration being directly related to the degree of DNA fragmentation. DNA amplification was carried out using specific primers and tritiated nucleotides. To characterize the degree of damage to DNA we used “coefficient of fragmentation”, *i.e.*, the ratio of total radioactivity of all “tail” fractions to that of the “head”.

### 3.2. Choice of Bioprotectors

A review of the literature on the mechanisms of toxic and genotoxic action of copper combined with our experience in the testing of various bioprotectors against other intoxications (summarized in [50]) allowed us to choose substances for estimating their possible protective action at subchronic intoxication with nano copper oxide, namely:

- Glutamate as an effective cell membrane stabilizer through the intensification of ATP synthesis under exposure to the damaging action of various cytotoxic particles (e.g., [7,48,49,50,51] and, at the same time, as one of the three precursors of glutathione, a powerful cell protector against free radicals.
- The other two precursors of glutathione: Glycine and cysteine (the latter in a highly active and metabolically well available form of *N*-acetylcysteine).
- Other components of the organism’s anti-oxidant system (vitamins А, Е, and С, and selenium).
- Trace elements, which are physiological antagonists of copper (zink, iron, molybdenum, manganese).
- ω-3 polyunsaturated fatty acids, whose intracellular derivatives are eicosanoids that activate DNA replication and thus play an important part in its repair, which was demonstrated by us earlier under exposure to various genotoxic agents (e.g., [7,51])
- Pectin enterosorbent as an agent that prevents the re-absorption of the metal excreted into the intestines with bile.

The doses and administration methods of these bio-protectors are given in Table 5.

**Table 5:** Doses and the mode of administration of the bioprotectors tested in our experiment.

Bioprotectors (See the Text for Explanation)	Estimated Dosage and the Mode of Administration
Sodium glutamate	800–900 mg/kg (as a 1.5% drink instead of water)
Apple pectin	1 g/kg (added to the fodder)
Glycine	12 mg per rat (added to the fodder)
Acetyl-cysteine	30 mg per rat (added to the fodder)
A commercial fish oil rich in vitamin A and omega 3 rich PUFA	1 mL per rat (by gavage)
**Multivitamin-Multimineral Preparations (Added to the Fodder) Supplying**	
Vitamin B12	0.15 mcg per rat
Vitamin C	4.4 mg per rat
Vitamin E	0.84 vg per rat
Iron	0.6 mg per rat
Selenium	5.8 mcg per rat
Zink	1.25 mg per rat
Molibdenum	3.75 mcg per rat
Manganese	16.7 mcg per rat

We gave glutamate to rats as 1.5% solution instead of the drinking water ad libitum. “An Amber Dew” (by “Ecco-Plus Ltd.”, Zhukovskiy, Russia), a fish oil preparation rich in PUFA pertaining mainly to the ω-3 group (24%) was administered through gavage at a dose of 1 mL per rat. The apple pectin enterosorbent (by “Promavtomatika Ltd.”, Belgorod, Russia) was added to the rats’ fodder in a quantity corresponding to a dose *ca.* 1000 mg/kg body weight. Other commercial preparations of amino acids, vitamins, and minerals (by “UfaVITA”, Ufa, Russia) available as tablets were crushed and added to another portion of the fodder in quantities corresponding to recommended daily intake of these micronutrients by rats (where such recommendations were known only for humans, a recalculation to the rat’s nutritional requirement was made based on the species’ standard metabolism ratio).

Taking into account that the standard balanced food presumably meets the normal nutritional requirements of a rat, we assumed that additional intake of the above-listed bioactive substances would meet the increased needs connected with molecular mechanisms of copper toxicity. Nevertheless, it had to be checked whether or not such “overloading” with them would evoke any unfavorable effects. That is why in our subchronic experiment one group of rats was administered the same BPC but not exposed to any toxicant.

## 4. Conclusions

Although engineered copper nanoparticles with this or that Cu to O ratio in their composition were demonstrated to produce toxic effects in a number of published studies, the literature lacks any *in vivo* chronic or, at least, subchronic toxicity characterizations of these effects, and there have been virtually no attempts to enhance the organism’s resistance to their impact. Thus, the totality of the currently available literature data is not sufficiently relevant to the occupational and environmental health problems. Specifically, it should be kept in mind that at copper smelters and refineries, as well as in the manufacture of copper alloys, the workroom and ambient air is polluted with condensation aerosols a significant fraction of which is represented by copper-containing particles <100 nm. The copper oxide nanoparticle suspensions prepared by us and used in our subchronic animal experiment can be deemed adequate as a model of this environmental toxicant.

The results obtained demonstrate that copper oxide nanoparticles are adversely bio-active in all aspects considered in this study, their solubilization in biological fluids playing a presumably important role as a prerequisite for their toxicokinetics and toxicodynamics. A toxicological syndrome comprising accumulation of Cu in liver and brain, some decrease in serum ceruloplasmin level, marked damage to brain basal nuclei neurons, and anemia is known to be characteristic of the Wilson’s disease in humans, and we believe that in the animal experiments described in this paper it may be considered specific for copper chronic toxicity.

Marked damage to genomic DNA in cells of different organs suggests that these nanoparticles can trigger carcinogenesis and thus perhaps enhance cancer risks well-established to be associated with copper metallurgy due to its inevitable sinister satellites—arsenic and cadmium.

However, we have demonstrated (for the first time, to the best of our knowledge) that a “bio-protective complex” comprising pectin, a multivitamin-multimineral preparation, some amino acids and fish oil rich in ω-3 PUFA, attenuated systemic and target organ toxicity, as well as the genotoxicity of these nanoparticles.

In summary, judging by experimental data, actual exposures to nano-scale copper oxide particles can present a significant health risk, and further research into its management with the help of innocuous bioprotectors seems to be justified.
